# Polynomial Supertree Methods Revisited

**DOI:** 10.1155/2011/524182

**Published:** 2011-12-21

**Authors:** Malte Brinkmeyer, Thasso Griebel, Sebastian Böcker

**Affiliations:** Department of Computer Science, Friedrich Schiller University, 07743 Jena, Germany

## Abstract

Supertree methods allow to reconstruct large phylogenetic trees by combining smaller trees with overlapping leaf sets into one, more comprehensive supertree. The most commonly used supertree method, matrix representation with parsimony (MRP), produces accurate supertrees but is rather slow due to the underlying hard optimization problem. In this paper, we present an extensive simulation study comparing the performance of MRP and the polynomial supertree methods *MinCut Supertree*, *Modified MinCut Supertree*, *Build-with-distances*, *PhySIC*, *PhySIC_IST*, and *super distance matrix*. We consider both quality and resolution of the reconstructed supertrees. Our findings illustrate the tradeoff between accuracy and running time in supertree construction, as well as the pros and cons of voting- and veto-based supertree approaches. Based on our results, we make some general suggestions for supertree methods yet to come.

## 1. Introduction

In recent years, supertree methods have become a familiar tool for building large phylogenetic trees. Supertree approaches combine input trees with overlapping taxa sets into one large and more comprehensive tree [1]. Systematists have probably used informal supertree methods since the beginning of systematics itself, pasting together hierarchically nested taxa. Since the introduction of the term *supertree* and the first formal supertree method [2], there has been a continuous development of such methods, see for example Bininda-Emonds [3]. In contrast to the combination of input trees, the combination of input datasets into a matrix of *characters*, and subsequent analysis of this matrix under standard reconstruction criteria is called the supermatrix approach, see for example de Queiroz and Gatesy [4]. Both approaches hold the promise to reconstruct phylogenies for large clades of the tree of life, for example, Bininda-Emonds et al. [5] reconstructed a supertree on 4510 taxa for the entire group of mammals.

It is well known that inferring optimal trees from sequences under the maximum likelihood (ML) [6] and the maximum parsimony (MP) criterion [7] are computationally hard problems, so we have to rely on heuristics that cannot guarantee to find the optimal solution. Even for a moderate number of species, the sheer size of the tree space prohibits searching for optimal trees under these criteria. Although some studies with thousands of taxa have been reported in the literature, they mostly intend to investigate new concepts of implementation and computation, for example, an ML analysis of 10,000 taxa on a parallel computer [8]. It remains unclear how close the resulting tree is to the optimal one, considering the hardness of the problem together with the enormous number of trees to be searched. For 10,000 taxa there exist 8.0 · 10^38658^ unrooted binary phylogenetic trees, a number much larger than the number of atoms in the observable universe. Clearly, supertree approaches with polynomial running time can be advantageous with respect to running time. But supertree methods have certain additional advantages over standard phylogenetic reconstruction methods [9]. They allow us to combine heterogeneous data sources, such as DNA hybridization data, morphological data, and protein sequences.

Current supertree methods can roughly be subdivided into two major families: matrix representation (MR) and polynomial, mostly graph-based methods. The former encode the inner vertices of all input trees as partial binary characters in a matrix, which is analyzed using an optimization or agreement criterion to yield the supertree. Matrix representation with parsimony (MRP) [10, 11], the first matrix-based method, is still by far the most widely used supertree method today. Other variants have been proposed using different optimization criteria, for example, matrix representation with flipping (MRF) [12] or matrix representation with compatibility (MRC) [13].

All MR methods have in common that the underlying optimization problems are computationally hard [7, 12, 14], and heuristic search strategies have to be used. As for ML and MP, it is unclear how close the resulting tree is to the optimal one. 

Graph-based methods make use of a graph to encode the topological information given by the input trees. This graph is used as a guiding structure to build the supertree top-down from the root to the leaves. Graph-based methods often use local optimization criteria and lack an explicit global criterion [15]. The first graph-based supertree method was the Build algorithm [16], which is only applicable to nonconflicting input trees and thus only of limited use, because “as most systematics know, phylogenies usually conflict with each other” [3]. This led to the development of the *MinCut Supertree* algorithm (MC) [17] and a modified version, *Modified MinCut Supertree* (MMC) [15], that are able to construct a supertree if the input trees are conflicting. The *Build-with-distances* algorithm (BWDs) [18] is the first graph-based method that uses branch length information from the input trees to build the supertree. All of these methods have in common that they apply a *voting* procedure to deal with conflicts among the input trees. They resolve conflicts by asking the input trees to vote for a clade in the supertree, such that, to a certain extent, the most frequent alternative is chosen.

In case of conflicting input trees, voting methods such as MRP can infer supertrees in which clades are present that contradict each of the input trees [19–21]. To which extent and under which circumstances these unsupported and undesired novel clades occur is subject of ongoing debate, see for example [20, 22]. Ranwez et al. [23] presented a graph-based method named *PhySIC*, which ensures that the reconstructed supertree satisfies two properties: it contains no clade that directly or indirectly contradicts the input trees, and each clade in the supertree is present in an input tree or is collectively induced by several input trees. Supertree methods guaranteeing the first property are called *veto* methods and, for highly conflicting and/or overlapping input trees, tend to produce unresolved supertrees. Scornavacca et al. [24] presented a modified version of *PhySIC*, *PhySIC_IST*, that tries to overcome this drawback by proposing nonplenary supertrees (i.e., supertrees that do not necessarily contain all taxa from the input trees), while still assuring the properties mentioned above. *PhySIC_IST* works in a stepwise fashion, iteratively adding leaves to a starting tree consisting of two nodes. Unlike the above mentioned polynomial supertree methods, the super distance matrix (SDM) method [25] does not descend from the Build algorithm. SDM modifies source distance matrices in a way that their “topological message” is not modified. These modified matrices are averaged and analyzed by distance-based tree-building algorithms. In contrast to MR methods, all mentioned methods (MC, MMC, BWD, *PhySIC*, *PhySIC_IST*, and SDM) have polynomial running time.

To build even larger portions of the tree of life, a promising approach for the future is the use of supertree methods as part of divide-and-conquer metatechniques such as disk covering [26–28]. Here, the basic idea is to break down a large phylogenetic problem into smaller subproblems that are computationally easier to solve, because of the lower number of taxa and the smaller evolutionary distance between them. Subresults are combined via supertree methods to find an answer for the initial, global problem. By using preferably fast (polynomial-time) and accurate supertree methods that compute resolved supertrees, “a divide-and-conquer strategy promises gains in both accuracy and speed compared to a conventional phylogenetic analysis” [9].

As an increasing number of supertree methods is available, studies using either simulated or empirical data are needed to compare the behavior and performance of the methods undervarious conditions. In simulation studies, results of different methods can be compared to a known model tree and, thus, the methods can be compared at an absolute scale, whereas empirical datasets may offer a more realistic setting, however, the true tree is usually not known. Several simulation studies focusing on different aspects of the investigated supertree or supermatrix methods have been carried out, see for example [22, 29–33]. But only recently, these studies started to provide relevant comparisons of alternative approaches. In this paper, we focus on a particular subset of supertree construction methods: we compare the accuracy of the by far most used and studied MRP method as representative of the MR-based family of supertree methods, with the mentioned polynomial-time supertree methods MC, MMC, BWD, *PhySIC*, *PhySIC_IST*, and SDM. MR-based methods have been shown to be accurate and usually generate highly resolved supertrees, but require long running times; graph-based methods are usually swift but possibly less accurate and, in case of *PhySIC* and *PhySIC_IST*, also possibly less resolved.

We present a large-scale simulation study conducted to compare the accuracy and the resolution of the computed supertrees. Additionally, we explore new variations of BWD, trying to improve its performance. Our simulation study follows the established general scheme to assess the performance of supertree methods: (1) construction of a model tree under a Yule process, (2) simulation of DNA alignments along that tree, (3) random deletion of a proportion of taxa, (4) reconstruction of trees by ML, (5) construction of a supertree from the inferred ML trees, and, finally, (6) comparison of the supertree to the model tree using distance and similarity measures, plus evaluation of its resolution. Our results demonstrate that the BWD and the *PhySIC_IST* method perform significantly better than MC and MMC. With respect to the accuracy of the reconstructed supertree, these two methods are sometimes even comparable with MRP. Unfortunately, *PhySIC_IST* often outputs nonplenary supertrees that exclude a large percentage of taxa, in some cases more than 50%. By considering the resolution of the supertrees, our findings illuminate not only the tradeoff between accuracy and running time in supertree construction, but also the pros and cons of voting and veto approaches.

When methods using branch lengths are applied in real-world studies, branch length from different input trees have to be reconciled. To this end, we outline a robust estimator for average multiplicative constants of branch lengths from different input trees, that combines the advantages of mean and median computation. Based on our results, we make several suggestions for the future of supertree construction.

This paper is organized as follows: in the next section we recall some basic terminology. In Section 3, we outline the methods under consideration and detail our variations of BWD. The use of branch length from simulated data and real-world data is discussed in Section 4, followed by a section describing our simulation protocol in detail. In Section 6 we present our results, which are discussed in Section 7.

## 2. Preliminaries

Our notations and definitions mainly follow Semple and Steel [34]. A *graph*, denoted *G* = (*V*, *E*), is a structure consisting of a set *V* of *vertices*, and a set *E*⊆{{*x*, *y*} : *x*, *y* ∈ *V*} of connections called *edges*. A graph is *simple* if it has no loops or parallel edges, and it is called *weighted* if each edge *e* ∈ *E* has a weight *w*(*e*) assigned to it. Given *E*′⊆*E*, we let *G*∖*E*′ denote the graph obtained from *G* by deleting all edges in *E*′. If *G*∖*E*′ is disconnected, *E*′ is called a *cut set* of *G*. In a weighted graph, *w*(*E*′) : = ∑_*e*∈*E*′_ *w*(*e*) is the *cut weight* of *E*′. A cut set of minimum weight is called a *minimum cut.* A *connected component C*_*i*_ of a graph is a maximal set of connected vertices, that is, for any pair of vertices *x* and *y* there is a path from *x* to *y*.

A tree *T* = (*V*, *E*) is a simple, connected graph with no cycles. A vertex *v* ∈ *V* is *internal* if the degree of *v* is greater than one, otherwise *v* is a *leaf*. For a given tree *T*, *L*(*T*) denotes the set of leaves of *T*. If *L*(*T*) = *X*, and *T* has one distinguished internal vertex, denoted *root*, and no vertex but the root may have degree two, then *T* is called *rooted phylogenetic tree (on X*). As most of the supertree methods under consideration in this paper require rooted trees as input, we neglect the unrooted case. For brevity, we often use the terms “rooted phylogenetic tree” and “tree” synonymously in the following. In a rooted phylogenetic tree, the *out-degree* of the root is simply its degree, whereas the out-degree of all other vertices is the degree minus one. A tree is *binary* if there is no vertex with out-degree larger than two, otherwise, the tree contains *polytomies*. In a tree *T* with weighted edges, the *path length* between two vertices *x*, *y* ∈ *V*, denoted pl(*x*, *y*), is the sum of weights of all edges between *x* and *y*.

Let *T* be a tree on *X*. An element of *X* is a *descendant* of an internal vertex *v* of *T* if the path from this element to the root contains *v*. Given a particular internal vertex *v*, a *clade* of *T* is a subset of *X* that consists of all elements of *X* that are descendants of *v*. For a given subset *X*′⊆*L*(*T*) we refer to the unique vertex of *T* that is the *last common ancestor* (also referred to as *least common ancestor*) of *X*′ in *T* as lca(*X*′).

Given *X*′⊆*L*(*T*), we construct the *restriction* of *T* to *X*′, denoted *T* | *X*′, by first finding the minimal subtree of *T* containing  *X*′, and then suppressing all vertices of degree two except for the root. 

For a set of phylogenetic trees *𝒯* = {*T*_1_,…, *T*_*x*_}, let *L*(*𝒯*) denote the set of leaves that appear at least in one tree. Let *T*_1_ and *T*_2_ be two trees with *L*(*T*_1_)⊆*L*(*T*_2_). The tree *T*_2_ *displays T*_1_, if *T*_2_ | *L*(*T*_1_) is a *refinement* of *T*_1_, that is, *T*_1_ can be obtained from *T*_2_ by contracting edges. Informally, *T*_2_  displays *T*_1_ if, up to polytomies, all the ancestral relationships of *T*_1_ are preserved in *T*_2_. For a set of trees *𝒯* with possibly overlapping leaves, we say that *𝒯* is *compatible* if there exists a tree T* on *L*(*𝒯*) that displays every tree *T*_*i*_ ∈ *𝒯*. Otherwise, *𝒯* is *incompatible*. When *𝒯* is incompatible, it is desirable to find a tree T* over *L*(*𝒯*) that minimizes some objective function measuring the incompatibility. Then T* is called a *supertree* and the problem of finding T* is called the *supertree problem*. Since biological data is incompatible for a range of reasons, including sampling errors, inaccuracies, or biases in tree building algorithms, incompatible input trees are what has to be expected in reality.

A *triplet* is a binary tree with three leaves. The triplet with leaves *x*, *y*, *z* is denoted by *xy* | *z* if the path from *x* to *y* does not intersect the path from *z* to the root. Given a tree *T*, rt(*T*) denotes the set of all triplets of *T*. For a collection of trees *𝒯*, rt(*𝒯*) denotes the set of all triplets of *𝒯*.

## 3. Methods under Consideration

### 3.1. Build and MinCut Supertrees

The first graph-based supertree method is Build [16], which was originally developed in the context of relational databases. Build is an all-or-nothing algorithm that returns a supertree only if the input trees are compatible. The supertree is constructed from the root to the leaves guided by a graph that we will call *Build graph*. In the first iteration of the algorithm, all leaves from the input trees are used as vertices in the Build graph. Two vertices *x*, *y* are connected in the Build graph if there is a triplet *xy* | *z* in at least one of the input trees. The resulting connected components correspond to the clades beneath the root of the supertree. Then, the algorithm is recursively called on the connected component.

The MinCut Supertree algorithm (MC) by Semple and Steel [17] was the first extension of Build capable of returning a supertree if the input trees are not compatible. The incompatibilities are resolved by deleting a minimal amount of information present in the input trees in order to allow the algorithm to proceed. This is done by disconnecting a modification of the Build graph whenever it consists of only one connected component. Page [15] presented a modified version of MC, that uses more information from the input trees. Using a modified graph construction, the Modified MinCut Supertree (MMC) algorithm ensures to incorporate all clades from the input trees with which no single tree directly disagrees.

### 3.2. Build-with-Distances Supertrees

Willson [18] presented the Build-with-distances (BWDs) algorithm that, in addition to the branching information in the input trees, uses branch lengths to build the supertree. The method follows the same recursive schema as Build, MC, and MMC. The main observation underlying the BWD algorithm is that branch lengths may carry phylogenetic information, such as an estimated number of mutations. In a biological application, using branch length is apparently only justified if these are comparable amongst the input trees, see Section 4.

For two leaves *x*, *y*, the BWD algorithm employs the distance from *x* to the last common ancestor of *x* and *y*, denoted as *λ*(*x*, *y*). Note that *λ* is not symmetric. If more than one input tree contains both *x* and *y*, the average of these distances is used. The graph used by the BWD method, called *BWD graph* in the following, extends the Build graph by additional edges. These edges arise through examination of the branch lengths in the input trees: Two leaves *x* and *y* may be in one input tree, and *x* and *z* are in another input tree, but no input tree contains all three leaves. If *λ*(*x*, *y*) < *λ*(*x*, *z*), the BWD graph will still contain an edge {*x*, *y*}. In case the BWD graph is connected, edge weights are determined using *support functions*, which estimate the evidence that two taxa should be in the same clade of the supertree. Let *U* be a nonempty subset of *L*(*𝒯*), corresponding to the connected component from a previous level of the algorithm. Initially, we have *U* = *L*(*𝒯*). For a triplet *xy* | *z* with *x* ≠ *y*, we define the *primary evidence* as *p*(*x*, *y*, *z*)∶ = max⁡{0, *λ*(*x*, *z*) − *λ*(*x*, *y*)}. In case {*x*, *y*} or {*x*, *z*} are not together in an input tree, we set *p*(*x*, *y*, *z*)∶ = 0.

Willson [18] introduced four support functions, namely, the *primary support function* (SP), the *confirmed support function* (SC), and the *accumulated primary support function* (SAP). Finally, the *accumulated confirmed support function* (SAC) is defined as

(1) $$\text{SAC}\left(x,y,U\right)∶=\overset{}{\underset{z\in U}{\sum }}\min\left\{p\left(x,y,z\right),p\left(y,x,z\right)\right\},$$

where again *U* is the clade from the previous step of the algorithm. In our simulations we find that supertrees built using SAC consistently outperform those built using the other support functions.

In contrast to the minimum cut approach of MC and MMC, the BWD method uses the *bisection method* to disconnect the BWD graph in case it consists of one component. We determine the minimum threshold *θ* so that, after removing all edges *e* with weight *w*(*e*) ≤ *θ* from the BWD graph, the resulting graph is disconnected. Different from MC and MMC, BWD does not guarantee to return the parent tree in case the input trees are compatible. This behaviour is intended, as distance information in the input tree might hint towards incompatibilities not observable in the topological structures of the input trees.

In some sense, the support functions suggested in [18] are conservative; for example, bounding the primary evidence to zero is somewhat arbitrary. We investigated several modifications of support functions from [18]. In our simulations, we found that supertrees built using the SAC and SAC_max⁡_ support functions, the later being a modification of the accumulated primary support function SAC, consistently outperform those built using all other support functions. To this end, we defer further details, and we will concentrate on these two support functions in our evaluation. In detail, we define SAC_max⁡_ as:

(2) $$\text{SA}{\text{C}}_{\max}\left(x,y,U\right)∶=\overset{}{\underset{z\in U}{\sum }}\max\left\{p\left(x,y,z\right),p\left(y,x,z\right)\right\}.$$

### 3.3. *PhySIC* and *PhySIC_IST* Supertrees

Following [23], supertree methods apply either a *voting* or *veto* procedure. Voting methods resolve conflicts by using an optimization criterion in order to select between different possible topologies [35]. When input trees conflict, voting methods such as MRP and MRF can infer supertrees in which clades are present, that are contradicted by each of the input trees [19–21]. Veto methods are more conservative in handling conflicts among the input trees: the inferred supertree has to respect the phylogenetic information of each source tree and is not allowed to contain any clade that is contradicted by one of the input trees. Thus, conflicts among the input trees are removed [35] which resultd in multifurcations in the supertree.

Scornavacca et al. [24] presented *PhySIC_IST*, a modification of the *PhySIC* algorithm [23], aiming to circumvent a main drawback of veto supertree methods. These tend to return highly unresolved supertrees if the input trees imply a high degree of incompatibility or do not have a high degree of overlap. To overcome this shortcoming, *PhySIC_IST* modifies the original approach by allowing nonplenary supertrees (i.e., supertrees that do not necessarily contain all taxa present in the input trees). *PhySIC_IST* uses a preprocessing step called source tree correction (STC), which analyses conflicting triplets among the input trees. The extent with which STC corrects the input trees is determined by a user-defined parameter *c* ∈ [0,1]. For *c* = 1 *PhySIC_IST* behaves like a pure veto method, while for *c* = 0 it mimics a voting method.

### 3.4. Super Distance Matrix (SDM) Supertrees

Basis for the SDM method by Criscuolo et al. [25] is the average consensus procedure (ACS) [36]. The first step of ACS is to compute distance matrices corresponding to the path-length in the input trees. After standardizing each input matrix, ACS computes the average of the standardized matrices which is used to build the distance supermatrix. The presented standardization procedure relies on dividing all distances in each matrix by the maximum distance in that matrix. This distance supermatrix is then analyzed using a least-squares method. Similar averaging methods are presented to generate the distance supermatrix directly from sequences and gene trees. Other standardizing methods have been explored, but seem to be inaccurate for more than two trees [37]. Similar to ACS, the distance-based method SDM uses a more involved standardization procedure and is able to use both sequences and gene trees as input (See [25] for details). The possibly still incomplete super distance matrix is then processed using MRV* (a variant of minimum variance reduction), BioNJ* or NJ* (variants of neighbor-joining) [38].

### 3.5. Matrix Representation with Parsimony (MRP)

MRP encodes the inner vertices of all input trees as partial binary characters in a matrix, then analyzes the resulting matrix under the parsimony criterion. Two different coding schemes have been suggested for the matrix representation, namely the Baum-Ragan (BR) and the Purvis (PU) coding scheme. Furthermore, two kinds of parsimony can be used: reversible Fitch parsimony and irreversible Camin-Sokal parsimony. MRP, with Baum-Ragan coding and Fitch parsimony, is commonly used and generally accepted as the standard method for supertree construction. Using BR coding, we add a column to the matrix representation for each interior edge of each input tree. Here, “0” and “1” encode taxa on either side of the edge, whereas missing taxa are encoded as “?”. In case of rooted trees, the clade is encoded “1” and the root is encoded “0” (See [10, 11, 39] for details). The MRP method then tries to find the most parsimonious tree for the matrix representation. Using reversible Fitch parsimony [40], we assume character changes to be undirected, so that both 0 → 1 and 1 → 0 flips are allowed along an edge. Unfortunately, the underlying Maximum Parsimony problem is computationally hard [7].

## 4. Simulated versus Real-World Data

Branch length from the input trees in a simulation study is arguably a best-case scenario for the BWD method, as the trees are simulated using the same parameters (see Section 5 for details). Nevertheless, if two taxa *x* and *y* cooccur in two input tree *T*_*i*_ and *T*_*j*_, typically it is not true that *λ*_*i*_(*x*, *y*) = *λ*_*j*_(*x*, *y*). Willson's as well as our implementation of the BWD algorithm merely averages the different values *λ*(*x*, *y*) obtained from different input trees, which works well in our simulation. If the BWD method is going to be used in a real-world study, branch length from different input trees have to be comparable. Clearly, careful studies on how to reconcile different distances have to be performed before applying BWD. In the following we outline ideas about how to reconcile branch-length from real-world data. First, we can compute a pairwise distance matrix for all taxa in the supertree, using either multiple alignments or pairwise alignments. We have different length estimates from different multiple datasets that we can normalize by finding a multiplicative constant for each dataset such that the sum of squared differences is minimized. Alternatively, we can use a linear program for this purpose, minimizing the maximum absolute difference. Finally, we can find multiplicative constants by a robust estimator. This results in a pairwise distance matrix *D*(*t*, *t*′) for all taxa *t*, *t*′.

We can then normalize branch lengths in each tree using either the largest distance in each tree, or all distances in the trees. Again we can minimize the sum of squared errors, use a linear program to minimize the maximum absolute error, or by a robust estimator for the multiplicative constant *c* such as median or *α*-trimmed mean. We exemplary show how to perform the latter. Let *S*⊆{1,…, *n*} denote the set of taxa in the input tree *T*, and let *D*_*T*_ be the pairwise distances for all *t*, *t*′ ∈ *S*. Let

(3) $$C∶=\left\{\frac{D\left(t,t^{\prime }\right)}{D_{T}\left(t,t^{\prime }\right)}:t,t^{\prime }\in S,t<t^{\prime }\right\}$$

be the set of multiplicative constants for all pairwise distances. We then compute the *α*-trimmed mean *c* from *C* for, say, *α* = 1/3. This is a robust estimator for the average multiplicative constant, combining the advantages of mean and median computation. Finally, we multiply all branch lengths in *T* by *c*. Note that normalizing branch lengths is not necessary if all branch lengths were computed under the same model of sequence evolution.

## 5. Simulation Study

**Figure 1:**
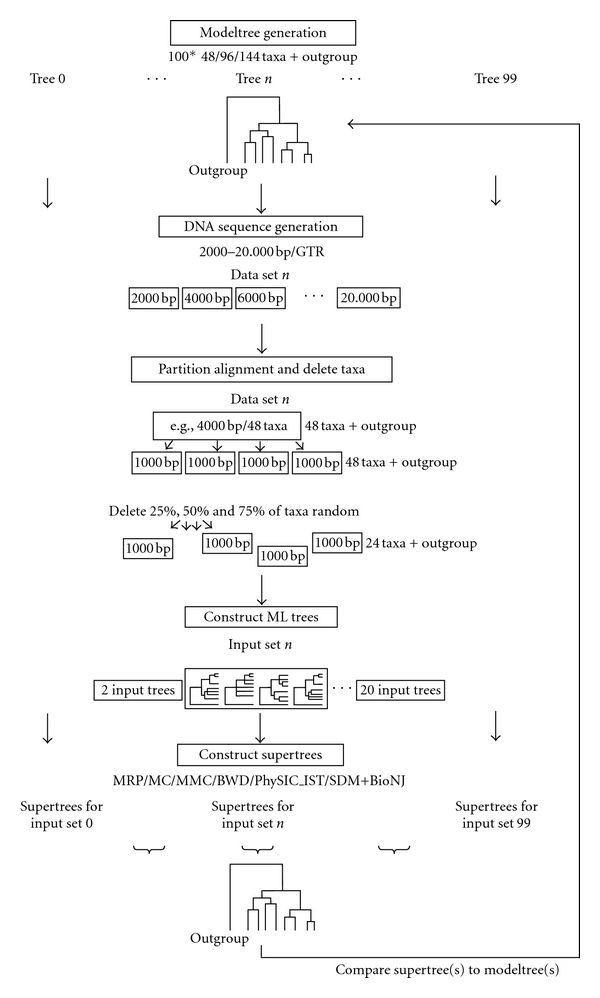
Simulation pipeline overview for 48, 96, and 144 taxa modeltrees.

We now present the layout of our large-scale simulation study, conducted to evaluate the accuracy and resolution of the methods MRP, MC, MMC, *PhySIC*, *PhySIC_IST*, BWD (including our new support function), and SDM. An overview of the simulation layout can be found in Figure 1. Each step is described in detail below. 

### 5.1. Generating Model Trees and DNA Sequences

We generated model trees according to a stochastic Yule birth process using the default parameters of the YULE_C procedure from the program r8s [41], with either 48, 96, 144, or 524 taxa. To deviate branch lengths on the trees from molecular clock, branch-specific rates of evolution were determined by drawing random normal variates (mean of 1.0 and standard deviation of 0.5, truncated outside of [0.1,2.0]) and multiplying by an overall tree-wide rate of substitution. These branch-specific rates are used to determine the branch-length by multiplying them with branch-durations obtained from the Yule process model. In each tree we set an additional outgroup since most of the supertree methods under investigation can only handle rooted trees. To determine the branch length for the outgroup, we proceeded as following. First, the taxon with the largest distance (*d*_max⁡_) to all other taxa is identified. If this distance exceeds 75% compared to all other distance relations, we shortened it accordingly. Then, we added an outgroup taxon with a branch length to the root that equals 1.25 × *d*_max⁡_. For model tree sizes of 48, 96, and 144, we generated 100 different model tree replicates and ten in case of 525 taxon model trees. The smaller number of replicates for 525 taxa model trees was due to the longer running times for this data set. Using Seq-gen v1.3.2 [42], nucleotide sequences were simulated along each of the model trees according to the general time reversible process (GTR) model [43] with parameters Lset Base = (0.3468,0.3594,0.0805), Rmat = (0.6750,27.9597,1.1677,0.4547,20.8760), gamma rate heterogeneity *α* = 1.1999, and PINVAR = 0.4954, taken from [44]. After sequence generation, we checked if the outgroup sequence has a larger distance to all other sequences than any two other sequences among each other. For each model tree we generated sequences ranging from 2000 to 20,000 base pairs in steps of 2000, yielding ten different “multiple sequence alignments” per model tree.

### 5.2. Generating Input Trees

The models of sequence evolution, implemented in Seq-Gen, assume that evolution is independent and identical at each site. Hence, we can partition the “multiple sequence alignment” with 2000–20 000 bases blocks, representing independent datasets. We chose an outgroup sequence/taxon to root the trees generated by maximum likelihood. In case of 48, 96, 144 taxon trees, we partitioned each alignment into blocks of 1000 base pairs each. From each alignment block, we randomly deleted 25%, 50%, or 75% of the sequences/taxa to simulate different taxa overlaps observed in real datasets. For each resulting alignment block, we inferred a maximum likelihood tree using RAxML v7.0.0 [45] with default parameters. This results in instances with 2 to 20 input trees corresponding to the same model tree. Summarizing, we have three different numbers of taxa; three different deletion ratios; ten different input sizes. For each of these 90-parameter combinations, we generated 100 instances. In case of 524 taxon model trees, we proceeded slightly differently from the described procedure: here, each alignment was partitioned into blocks of 500 base pairs, and 50% or 75% of the sequences/taxa were deleted. Again, a maximum likelihood tree was inferred for each resulting alignment block. In contrast to the 48, 96, 144 taxon model tree data sets, this results in instances with 4 to 40 input trees.

### 5.3. Supertree Construction

MRP supertrees were estimated using PAUP* 4.0b10 [46] with TBR branch swapping as heuristic search, random addition of sequences, and a maximum 10,000 trees in memory. In case of 48, 96, 144 taxon trees, the search time for a single MRP supertree run was delimited to 5 minutes and for 524 taxon model trees to one hour. Since we explicitly did not delete the outgroup sequence from the alignment, the root of each input tree is known. The strict consensus tree of all most parsimonious trees was used as the final MRP tree.

We computed MC as well as the BWD supertrees using our own implementations embedded in our software framework EPoS (http://bio.informatik.uni-jena.de/epos/) [47]. MMC trees were generated using Rod Page's implementation (http://darwin.zoology.gla.ac.uk/~rpage/supertree/). For the *PhySIC* and *PhySIC_IST* supertrees, we used the implementations provided by the authors of the corresponding papers (http://www.atgc-montpellier.fr/physic/binaries.php, http://www.atgc-montpellier.fr/physic_ist/) [23, 24]. We did not collapse any branches from the input trees on the basis a bootstrap threshold before applying the *PhySIC* and *PhySIC_IST* method (-b option). We mentioned above that *PhySIC_IST* offers a parameter for the STC preprocessing (-c option) that allows to tune the method from “veto-” to “voting-like.” We tested the method with parameter 0, 0.5, 0.8, and 1. We found that results for parameters 0 and 0.5 are similar, and so are those for parameters 0.8 and 1. Therefore, we report only results for parameters 0 and 1 below. In the following, we refer to these two parameter settings as *PhySIC_IST* 0 and *PhySIC_IST* 1. SDM + BioNJ* supertrees were computed using the implementation by Criscuolo et al. [25] (http://www.atgc-montpellier.fr/sdm/binaries.php), and PhyD* by Criscuolo and Gascuel [38] (http://www.atgc-montpellier.fr/phyd/binaries.php). We choose BioNJ* instead of FastME, because distance matrices in our simulation were incomplete. 

### 5.4. Measuring Accuracy and Resolution

To evaluate accuracy of the supertrees build by the different methods, we compared the supertrees to the corresponding model trees using different distances and similarity scores. Recall that *PhySIC_IST* usually computes nonplenary supertrees. In this case, we first restrict the model tree to the taxon set of the supertree. Consider a rooted tree where all but two taxa have been removed. Obviously, this tree will always coincide with the correct topology. So, we can obtain better distance and similarity scores by removing taxa, in particular those that we consider “doubtful.” In contrast, the MAST score (see below) will get smaller if we output a smaller tree. Hence, this approach favors *PhySIC_IST* for all distance measures except the MAST score, so *PhySIC_IST* results must be interpreted with some caution.

The Robinson-Foulds distance (*RF distance*) counts the number of clades that belong to only one of the two trees [48]. We normalize the RF distance by the number of internal nodes of both trees, yielding a value in [0,1].

Page [15] introduced the *triplet distance*, which is the rooted equivalent of the quartet metric [49]. For a triplet with three distinct taxa *x*, *y*, *z* there are five possible outcomes when comparing model tree and supertree. The triplet induced by *x*, *y*, *z* is resolved and identical in both trees (counted in same), or resolved and different (counted in diff); the triplet is resolved in the model tree *T*_1_ but not in the supertree *T*_2_ (counted in *r*_1_), or the other way round (counted in *r*_2_), or, the triplet is unresolved in both trees (counted in *x*). The triplet similarity can be defined as the number of shared resolved triplets, divided by the number of triplets that are resolved in at least one of the trees. We define the *triplet distance* as one minus the triplet similarity, so

(4) $$\begin{array}{cc} d_{\text{TR}}\left(T_{1},T_{2}\right) & ∶=1-\frac{\text{same}}{\text{same}+\text{diff}+r_{1}+r_{2}}\\ & =\frac{\text{diff}+r_{1}+r_{2}}{\text{same}+\text{diff}+r_{1}+r_{2}}. \end{array}$$

We note that in our simulation study, all our model trees are fully resolved and, hence, *r*_2_ = *x* = 0. We also note that the triplet type I error (false-positive rate) is very similar to the errors reported here, so we do not report type I errors individually.

The maximum agreement subtree score, or *MAST score* for short [50], counts the number of leaves of the maximum agreement subtree of the model tree and the supertree. The maximum agreement subtree was calculated using PAUP*. We normalize the MAST score using the number of leaves in the model tree. This indicates the fraction of the model tree that is recovered by the different methods.

We stress that each of these methods has its particular shortcoming: For example, Robinson-Foulds only evaluates perfectly matching clades, whereas the triplet distance favors top-level clades. Two phylogenetic trees of arbitrary size that share only two common clades, may get a triplet distance as low as 1/4, see the appendix. We do not want to assess the pros and cons of the three distance methods but, instead, propose to use them as a *relative* measure to assess the quality of supertrees computed by the different supertree methods.

Resolution was measured as the number of clades in the inferred supertree relative to the total number of clades on a fully binary tree of the same size (*n* − 2 for an unrooted tree, where *n* is the number of taxa). Resolution varies between 0 and 1, where 0 indicates a unresolved bush and 1 indicates a binary supertree.

Note that there are alternatives to measure the resolution of the supertrees, for example, based on the number of triplets in the supertree or based on the cladistic information content [51, 52].

## 6. Results

All computations were performed on a Linux cluster of AMD Opteron-2378, 2.4 GHz CPUs, with 16 GB of memory.

**Figure 2:**
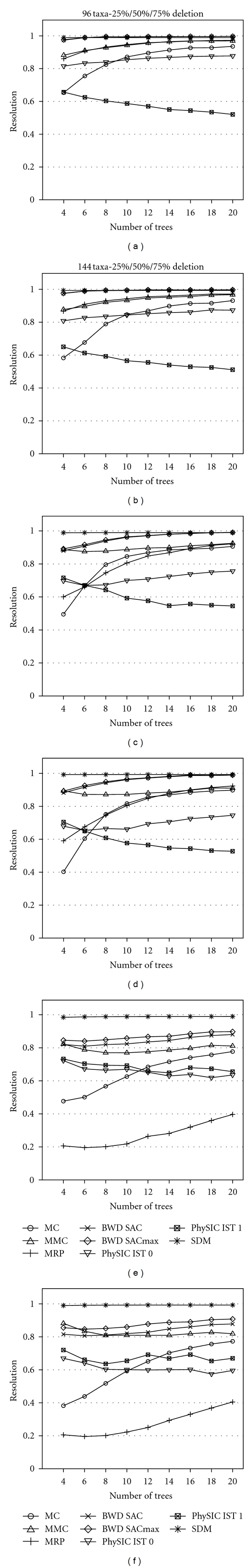
The left and the right column show the average resolution of the supertrees constructed from model trees with 96 and 144 taxa, respectively, and different taxon deletion ratio (top 25%, middle 50%, bottom 75%).

**Figure 3:**
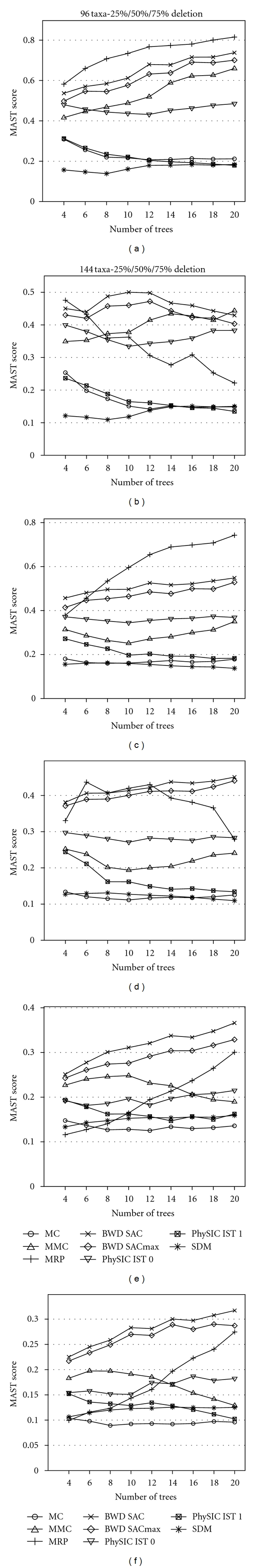
The left and the right columns show the MAST score of the supertrees constructed from model trees with 96 and 144 taxa, respectively, and different taxon deletion ratios (top 25%, middle 50%, bottom 75%). Note that the MAST score is a similarity score and not a distance.

**Figure 4:**
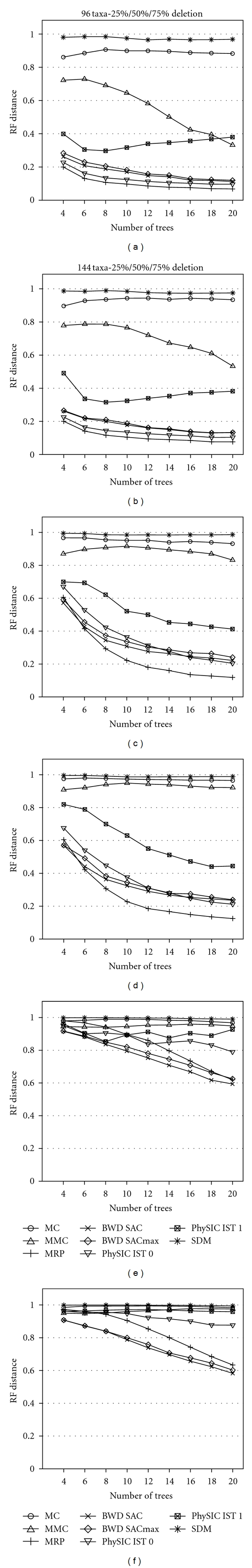
The left and the right columns show the average RF distance of the supertrees constructed from model trees with 96 and 144 taxa, respectively, and different taxon deletion ratios (top 25%, middle 50%, bottom 75%).

**Figure 5:**
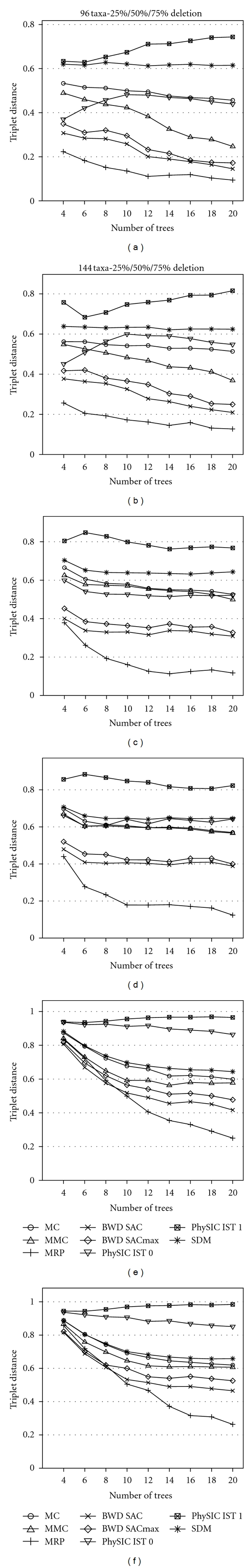
The left and the right columns show the average triplet distance of the supertrees constructed from model trees with 96 and 144 taxa, respectively, and different taxon deletion ratios (top 25%, middle 50%, bottom 75%).

**Figure 6:**
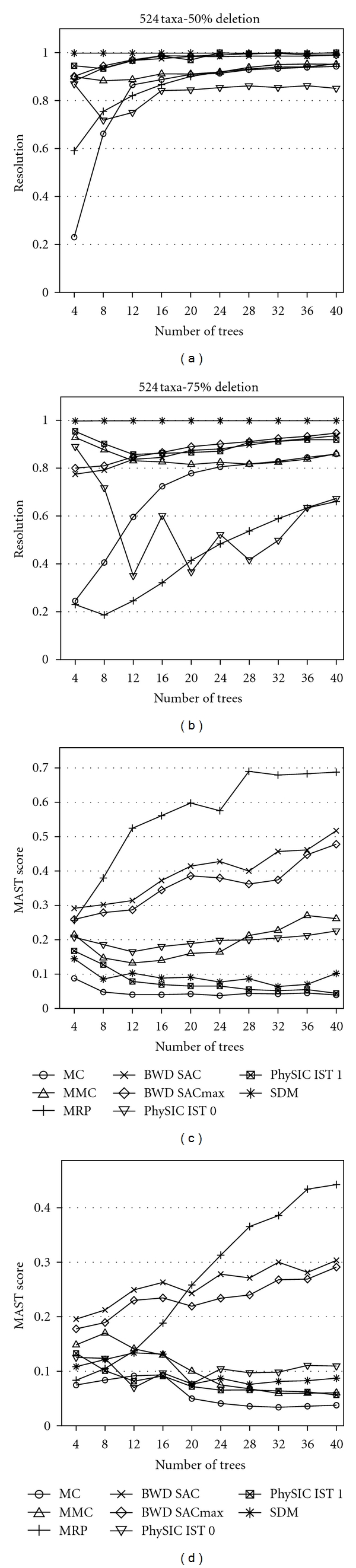
The upper row shows the average resolution of the supertrees constructed from model trees with 524 taxa and different taxon deletion ratios (left 50%, right 75%). The lower row shows the average MAST score of the supertrees constructed from model trees with 524 taxa and different taxon deletion ratios (left 50%, right 75%).

**Figure 7:**
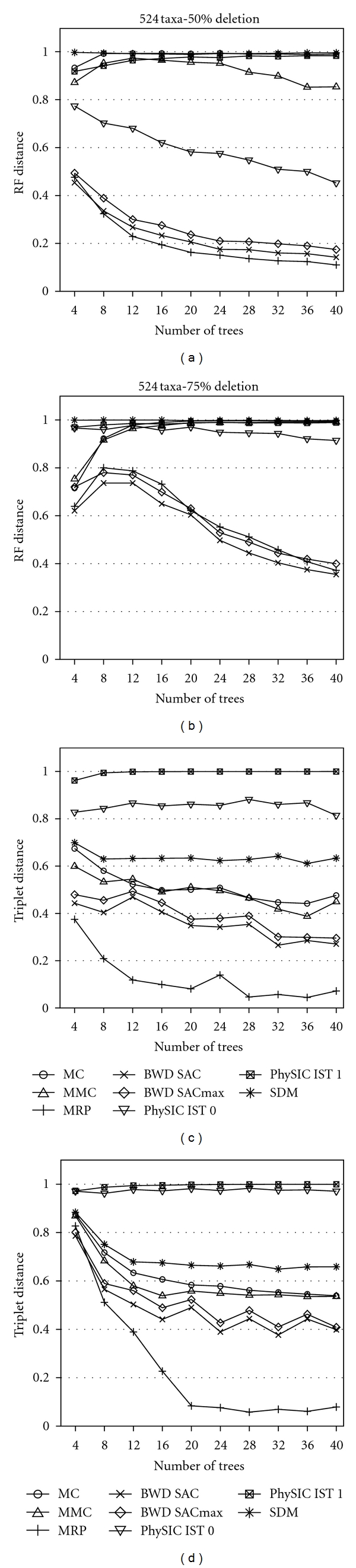
The upper row shows the average RF distance of the supertrees constructed from model trees with 524 taxa and different taxon deletion ratios (left 50%, right 75%). The lower row shows the average triplet distance of the supertrees constructed from model trees with 524 taxa and different taxon deletion ratios (left 50%, right 75%).

**Figure 8:**
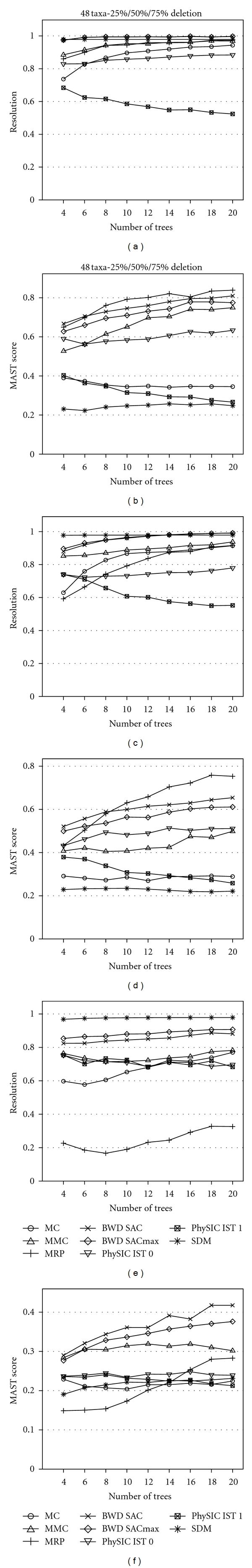
The left column of the figure shows the average resolution of the supertrees constructed from model trees with 48 taxa and different taxon deletion ratios (top 25%, middle 50%, bottom 75%). The right column shows the average MAST score of the supertrees constructed from model trees with 48 taxa.

**Figure 9:**
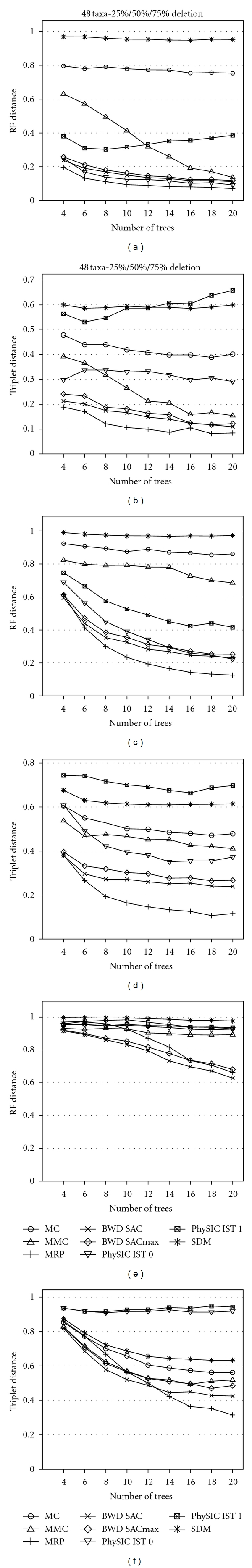
The left column of the figure shows the average RF distance of the supertrees constructed from model trees with 48 taxa and different taxon deletion ratios (top 25%, middle 50%, bottom 75%). The right column shows the average triplet distance of the supertrees constructed from model trees with 48 taxa.

The relative performance of the six supertree methods we studied with respect to supertree resolution, MAST score, RF distance and triplet distance is similar for different model tree sizes. Results of our simulation for 96, 144, and 524 taxa model trees can be found in Figures 2, 3, 4, 5, 6, 7 and for 48 taxa model trees in the appendix, Figures 8 and 9. We omitted standard deviation plots in these figures for the sake of readability. Running times of the polynomial supertree methods in case of 524 taxa model trees are listed in the appendix, Table 6. MC, MMC, BWD, and SDM+BioNJ* are relatively fast, the fastest being SDM+BioNJ*. In contrast, *PhySIC_IST* required more than 10 hours on this data set. MRP was constrained to a running time limit of one hour to reach a somewhat fair comparison. 

As mentioned above, *PhySIC_IST* can produce nonplenary supertrees, that is, supertrees that not necessarily contain all taxa from the input trees, see Tables 1, 2, 3, and 4 and in the appendix. As one would expect, the more conservative *PhySIC_IST* 1 excludes more taxa than *PhySIC_IST* 0, and the amount of excluded taxa increases with a higher deletion ratio. This can be explained by the decreasing degree of overlap between the input trees, which impedes the insertion of taxa while observing the PI property. For 145 taxa, 20 input trees, and 75% deletion ratio, almost three-fourths of the taxa were excluded.

Concerning the accuracy of the supertrees from the different methods, generally one would expect that results improve if more input data becomes available, as this helps to identify bogus information. Hence, in general the triplet distance and RF distance should decrease, whereas the MAST score should increase when more input trees are available to the supertree method. Below we discuss the observed patterns in more detail.

### 6.1. Resolution

Results concerning the resolution of supertrees from all methods under consideration can be found in Figures 2 and 6, upper row. It must be understood that a highly resolved supertree does not imply that this supertree is of good quality. In our evaluation, *PhySIC* mostly returns star trees even for 25% deletion ratio and all model tree sizes. For this reason, we decided to exclude the method from further investigation.

SDM+BioNJ* builds the most resolved supertrees for all model tree sizes, deletion ratios, and number of input trees, followed by the two variations of the BWD algorithm. In case of 25% deletion ratio, the BWD supertrees are also usually almost completely binary. For higher deletion ratios, the method requires more input trees to obtain higher resolved supertrees, but even for 75% deletion ratio, the method produces supertrees with a resolution consistently higher than 0.8. The behavior of MMC is similar to that of BWD, but the resolution is slightly worse in most cases. The MC method produces relative well-resolved supertrees at a deletion ratio of 25%. Higher deletion ratios and larger input trees have a significant negative influence on the resolution of the MC supertrees.

In case of 25% deletion ratio, MRP behaves similar to BWD, but with higher deletion ratio, the resolution of supertrees significantly decreases. At a deletion ratio of 75%, the resolution of supertrees produced by MRP is significantly smaller compared to all other methods. In case of 25% deletion ratio, the resolution of *PhySIC_IST* 0 supertrees increases with more input trees. The resolution is in general slightly lower compared to that of the supertrees produced by BWD, MRP, MMC. At deletion ratios of 50% and 75%, *PhySIC_IST* 0 produces less resolved input trees, as the number of input trees increases. For deletion ratios of 25% and 50%, the resolution of supertrees produced by *PhySIC_IST* 1 is worse compared to all other methods. The resolution decreases as the number of input tree increases. For a deletion ratio of 75%, *PhySIC_IST* 1 shows basically the same behavior, but the higher deletion frequency does not have such a negative effect regarding the resolution as it has for *PhySIC_IST* 0.

### 6.2. MAST Score

The MRP method performs better than all other methods in case of 25% and 50% deletion ratio and 48 or 96 taxa model trees. Here, MRP significantly benefits from a growing number of source trees. For 75% deletion ratio, the MAST scores of all methods under consideration are quite low, and MRP can only outperform some other supertree methods for a large number of input trees. For 145 taxa model trees and 25% and 50% deletion ratios, the MRP curve shows a peculiar zig-zag pattern. We repeated this experiment twice, but obtained similar results. This behavior might be explained by local maxima in which the heuristic gets stuck for larger input trees.

For 25% and 50% deletion ratios and 48 and 96 taxa model trees, both BWD methods are only outperformed by MRP. In both cases, the number of input trees has a positive effect on the MAST score. For 75% deletion ratio, both BWD variants outperform all other methods, although the MAST score is low for all methods. Again, for an increasing number of input trees, quality of BWD supertrees increases.

In most cases, *PhySIC_IST* 0 produces supertrees with a considerably better MAST score than MC, MMC, and *PhySIC_IST* 1, but the number of input trees has only a slightly positive effect on the MAST score. The MMC algorithm performs slightly better than *PhySIC_IST* 0 and 1 as well as the MC method in case of 25% deletion ratio. For a 25% deletion ratio MMC's MAST score increases with more input, in both other cases the score is relatively constant. The SDM+BioNJ* method produces supertrees with very low MAST score outperforming only MC for 50% and 75% deletion ratio.

### 6.3. RF Distance

In the case of 25% and 50% deletion ratio and all model tree sizes, MRP, the BWD variants, and *PhySIC_IST* 0 perform similarly and significantly better than *PhySIC_IST* 1, MMC and MC. The accuracy of the first three mentioned methods generally benefits from more input trees; in case of 25% deletion ratio, the accuracy increases significantly up to eight input trees and increases slower for more input trees, whereas in case of 50% deletion ratio the accuracy increases more constantly with the number of input trees. For 25% deletion ratio, the BWD variants are outperformed by *PhySIC_IST* 0 and MRP, for 50% deletion ratio BWD is slightly more accurate than *PhySIC_IST* 0, but MRP still outperforms both methods.

For 25% and 50% deletion ratio, *PhySIC_IST* 1 outperforms MMC and MC. In the former case, the accuracy of *PhySIC_IST* 1 increases only up to eight input trees and decreases slightly afterwards, whereas in the latter case a higher number of input trees has a positive influence on the accuracy. The accuracy of supertrees produced by the MMC method benefits from more input trees only in case of 25% and 50%, but in the former case the influence is more significant. The MC method performs worse compared to MMC. The number of input trees has no positive influence on the RF distance, which is constantly higher than 0.8 in most cases. For all model tree sizes and deletion frequencies, SDM+BioNJ* performs worst regarding the RF distance.

In the extreme case of 75% deletion, the RF distance of all methods is very high. Here, for 48 taxa model trees, *PhySIC_IST* 0 slightly outperforms MRP and the BWD variants. For both cases of larger model trees, the BWD variants outperform MRP as well as *PhySIC_IST* 0. But note that a RF distance of 0.6 and a resolution of the supertree of 0.9 implies that more than 54% of the true clades are present in the supertree.

### 6.4. Triplet Distance

For our evaluation, the triplet distance equals the triplet type II error, as the model tree is fully resolved. We also evaluated the triplet type I error but found that results do not differ significantly from those reported here; we omit the details.

MRP performs better than all other methods in case of 25% and 50% deletion ratio. For 75% deletion ratio, MRP outperforms the other methods only for ten and more input trees. A higher number of input trees consistently has a positive effect on the accuracy of the reconstructed supertrees.

Both BWD variants perform similarly for all parameters, although BWD SAC consistently produces more accurate supertrees. A large number of input trees has no significant positive effect on the accuracy. For 25% and 50% deletion ratio, the triplet distance of the reconstructed supertrees is slightly worse compared to the MRP supertrees. In case of 75% deletion ratio and few input trees the BWD variants are on par with MRP. Like BWD, the MC and the MMC algorithm perform similarly concerning all parameters, although the MMC algorithm generally performs better than MC. For both methods, the accuracy increases slightly with the number of input trees.

For all parameter settings, *PhySIC_IST* 1 produces significantly less accurate supertrees than all other methods. A higher number of input trees has no significant influence on the accuracy. *PhySIC_IST* 0 and SDM+BioNJ* produce better supertrees with respect to the triplet distance than *PhySIC_IST* 1. For deletion ratios of 25% and 50%, a higher number of input trees generally has a slightly positive effect on the accuracy. For a closer look at the performance of *PhySIC_IST*, we also compared the numbers of identical and different triplets in more detail, see the appendix.

## 7. Conclusion

We have presented a large-scale simulation study to assess and compare the accuracy and the resolution of supertrees constructed by polynomial supertree methods and the *de facto* standard supertree method MRP. Our results show that recent polynomial supertree methods can sometimes compete with the classical MRP approach while guaranteeing a significantly better running time, which did not exceed a few seconds for all polynomial methods. As mentioned in the introduction, one future approach to build larger clades of the Tree of Life might be a divide-and-conquer framework coupled with supertree methods, and “particular focus needs to be placed on developing fast supertree methods that yield accurate and well resolved solutions” [53].

The BWD method, which incorporates branch length information from the input trees, significantly enhances the graph-based approach concerning accuracy and resolution, without sacrificing short running times. We believe that further investigations concerning the use of branch length are needed, to make methods such as BWD applicable for real-world studies. On one hand, methods as the one proposed in Section 4 to reconcile different distances need to evaluated. On the other hand, different weighting schemes for edges in the BWD graph should be evaluated, which includes the use of bootstrap values from the input trees.

Veto approaches such as *PhySIC* have certain appealing properties but also certain drawbacks: the resolution of reconstructed supertrees rapidly decreases when there are too many conflicts among input trees, and/or small taxon overlap. In our simulation, *PhySIC* returned only star trees, a behavior that is certainly improved by collapsing branches from the input trees with low bootstrap support (the “-b” command line option in *PhySIC* program offers). In our simulation, we decided not to use this option but to use the unmodified input trees “as they are,” because we argue that a similar preprocessing would also enhance the performance of other methods. The effect of collapsing branches can be different for different methods, and *PhySIC* may benefit more from doing so, but we found this question to be beyond the scope of our evaluation study.

*PhySIC_IST*, in combination with STC preprocessing, significantly enhances the veto approach in terms of resolution and accuracy, but at the cost of taxa not being included in the supertree. The extent to which *PhySIC_IST* excludes taxa clearly depends on the used data set. The data used in our simulation represents a rather extreme case, and data that carefully selected by hand should be more suitable for *PhySIC_IST*. Furthermore, analysis of the triplet distance (or triplet type II error for our study) reveals that veto approaches can be too conservative, whereas voting approaches as BWD can be too decisive. For future polynomial supertree methods, a middle course between the two approaches seems desirable.

Simulation studies, as the one conducted here, have the general advantage that we can compare the reconstructed supertree with the true model tree. Our findings are generally in accordance with other supertree simulation studies. Both Eulenstein et al. [31] and Kupczok et al. [54] found that MRP outperforms MC and MMC. Eulenstein et al. [31] also found that matrix representation with flipping (MRF) shows a similar performance as MRP. Regarding SDM, we decided to use SDM+BioNJ* in our study because of running time considerations and incomplete distance matrices. Unfortunately, SDM+BioNJ* did not show competitive results. According to Buerki et al. [30] and Kupczok et al. [54], SDM+MW* performs much better than SDM+BioNJ*, but this again comes at the price of significantly increased running times. The BWD method has not been considered in previous simulation studies, but our results clearly indicate that BWD supertrees can be of good quality. Despite all developments in the field during the last 19 years, MRP must still be regarded a “gold standard” of supertree reconstruction; nonwithstanding its advanced age, there appears to be no method that clearly outperforms it. In particular, many methods (even those claiming polynomial running time) have running times that are unfavorable when compared to the highly developed MRP search heuristics. The empirical study of Buerki et al. [30] showed, somewhat surprisingly, that supertree quality for MC and MMC are comparable to MRP and MRF. Other methods such as average consensus or split fit showed much poorer performance. But according to Swenson et al. [55], the topological distance to source trees and the topological distance to the true is only weakly correlated, so empirical studies might be misleading.

Today, a divide-and-conquer approach to large scale using polynomial supertree methods as subprocedure has not fully been realized. Instead, we propose to use several of the supertree methods presented here for medium-sized studies with hundreds of taxa and tens of trees, and to manually compare the results. But if the sheer size of the problem makes it impossible to use matrix-representation methods such as MRP, novel polynomial-time methods such as BWD or variants of SDM can greatly improve the quality of results, compared to early methods such as MC or MMC. Although formal supertree methods have been around for a quarter of a century, our simulation also shows that there is still much room for improvement, and that novel ideas and methods can greatly improve the quality of constructed supertrees. 

## Figures and Tables

**Table 1:** The table shows average numbers of taxa excluded from supertrees build by *PhySIC_IST* from model trees of 48 taxa. Numbers are shown for different quantities of input trees, different deletion ratios, and different parameters of the STC process (-c option).

No. of input trees	25% deletion		50% deletion		75% deletion	
	*c* = 0	*c* = 1	*c* = 0	*c* = 1	*c* = 0	*c* = 1
2	4.53	4.53	3.77	3.77	4.91	4.91
4	0.83	5.6	7.29	12.22	10.2	10.23
6	0.36	2.17	5.36	13.96	15.39	15.41
8	0.23	1.45	3.77	11.26	18.89	19.9
10	0.13	4.23	2.69	8.66	22.13	23.22
12	0.17	1.38	2	7.49	23.15	24.65
14	0.16	1.62	1.48	4.94	25.76	27.18
16	0.07	2.22	1.08	4.44	25.35	27.43
18	0.16	2.05	1.1	4.17	25.52	28.87
20	0.05	2.34	0.89	3.15	26.41	28.64

**Table 2:** The table shows average numbers of taxa excluded from supertrees build by *PhySIC_IST* from model trees of 96 taxa. Numbers are shown for different quantities of input trees, different deletion ratios, and different parameters of the STC process (-c option).

No. of input trees	25% deletion		50% deletion		75% deletion	
	*c* = 0	*c* = 1	*c* = 0	*c* = 1	*c* = 0	*c* = 1
2	13.87	13.87	8.72	8.72	9.04	9.04
4	0.9	13.31	12.13	26.47	21.73	22.22
6	0.52	4.79	9.17	35.62	30.11	33.29
8	0.26	2.83	6.15	33.17	39.94	40.94
10	0.19	4.23	4.72	22.8	42.12	49.17
12	0.23	5.08	3.29	19.07	41.57	54.71
14	0.21	4.31	2.51	12.34	41.43	55.8
16	0.18	4.62	1.78	11.94	42.31	59.45
18	0.11	5.88	1.43	10.32	39.69	60.12
20	0.16	5.56	0.16	8.7	37.95	62.09

**Table 3:** The table shows average numbers of taxa excluded from supertrees build by *PhySIC_IST* from model trees of 144 taxa. Numbers are shown for different quantities of input trees, different deletion ratios, and different parameters of the STC process (-c option).

No. of input trees	25% deletion		50% deletion		75% deletion	
	*c* = 0	*c* = 1	*c* = 0	*c* = 1	*c* = 0	*c* = 1
2	30.62	30.6	15.59	15.59	11.41	11.41
4	2.2	36.8	18.74	53.2	31.67	34.04
6	1.52	12.24	13.8	67.92	48.41	55.25
8	1.75	8.1	10.6	58.8	57.18	70.5
10	1.76	6.23	7.13	48.53	64.1	86.8
12	1.54	7.17	4.77	38.61	61.28	95.15
14	1.64	7.26	4.07	28.72	63.2	99.17
16	1.5	9.96	3.21	22.62	62.26	103.78
18	1.6	9.94	2.91	16.35	57.4	104.02
20	2.21	9.11	2.86	17.46	59.57	105.81

**Table 4:** The table shows average numbers of taxa excluded from supertrees build by *PhySIC_IST* from model trees of 524 taxa. Numbers are shown for different quantities of input trees, different deletion ratios, and different parameters of the STC process (-c option).

No. of input trees	50% deletion		75% deletion	
	*c* = 0	*c* = 1	*c* = 0	*c* = 1
4	150.1	300.5	180	179.2
8	145.2	429.8	269.3	338.1
12	124.8	468.7	299.9	410.9
16	130.3	483.8	314.6	432.6
20	118.4	490	307.6	455
24	118.7	492.6	307.3	470.4
28	105.7	498.7	313.8	478.1
32	96	499.1	303.9	483.9
36	96	498.7	305.6	485.4
40	81.5	502.8	304.6	488.6

**Table 5:** The table shows the rounded average numbers of *d*, *r*_1_ and *s* (triplet error II) for *PhySIC_IST* 0, *PhySIC_IST* 1, BWD SAC, and MRP from model tree with 96 taxa. Numbers are shown for different quantities of input trees and different deletion ratios.

Method	No. of input trees	*d*			*r*_1_			*s*		
		25%	50%	75%	25%	50%	75%	25%	50%	75%
*PhySIC_IST* 0	2	79560	113096	143171	12445	3512	249	55435	30832	4020
	10	945	21989	132238	70095	55607	2226	76400	69843	12976
	20	769	5951	121243	63994	70944	6112	82676	70545	20085

*PhySIC_IST* 1	2	79560	113096	143171	12445	3512	249	55435	30832	4020
	10	17313	78432	138623	82097	39358	2321	48029	29650	6496
	20	22228	33872	139835	874160	79315	2426	37796	34253	5178

BWD SAC	2	53189	100519	13931	16	87	208	94246	46834	7841
	10	38105	48742	76237	1	7	92	109334	98691	71110
	20	21443	45524	61371	0	1	31	125997	101914	86037

MRP	2	45816	94097	137804	19002	11143	2776	82621	42201	6860
	10	12701	13033	32666	7273	10597	40878	127466	123810	73897
	20	10013	11712	15234	3882	5553	21545	133545	130174	110601

**Table 6:** Average running times (min:sec) of the polynomial supertree algorithms in case of 524 taxa model trees, different quantities of input trees, and different deletion ratios. Note that for this data set the running time of MRP was limited to 60 minutes, and all instances reached this time limit.

No. of input trees	BWD		SDM		MC		MMC		*PhySIC_IST*		*PhySIC_IST *	
									*c* = 0		*c* = 1	
	*d* = 50%	75%	50%	75%	50%	75%	50%	75%	50%	75%	50%	75%
4	18:17	5:54	1:16	0:12	3:28	0:33	0:33	0:04	3345:18	1026:55	2483:26	1736:14
8	35:33	26:12	1:48	0:20	36:27	5:22	18:36	0:54	3274:32	2386:27	1505:28	1741:35
12	41:45	46:35	2:28	0:30	60:21	17:18	72:24	4:58	2003:21	5732:01	1066:54	1659:21
16	44:22	44:56	3:35	0:43	67:21	29:02	94:04	14:13	1862:25	2916:44	914:09	1485:04
20	51:11	56:57	5:16	1:03	73:40	39:41	109:20	33:17	1427:29	4930:45	819:55	1341:26
24	46:30	48:54	7:24	1:12	80:41	43:15	113:33	51:01	1484:52	3527:05	825:59	1114:28
28	43:02	59:19	9:35	1:51	84:02	48:07	110:60	63:27	1632:38	3116:49	881:23	1073:11
32	29:21	62:07	12:38	2:24	87:26	51:49	96:40	79:33	1626:19	2518:55	894:34	982:14
36	29:04	59:56	17:03	2:46	91:41	57:12	95:23	89:09	1480:25	1883:58	898:09	922:50
40	20:51	64:33	20:07	3:24	93:31	59:52	89:43	101:59	1796:09	1683:19	897:45	859:44

## Appendix

*PhySIC_IST*: Number of Excluded Taxa
See Tables 1, 2, 3, 4.

Triplet Error II: Values of *d*, *r*_1_, and *s* for 96 Taxon Model Trees In order to compare the veto and voting approachs in more detail, it is interesting to compare the values the triplet error II is calculated from.  Table 5 shows the rounded average values of *d*, *r*_1_ and *s* for *PhySIC_IST* 0, *PhySIC_IST* 1, BWD SAC and MRP in case of 96 taxon model trees and two, ten and twenty input trees. The described behavior of the methods is similar for 48 and 144 taxa model trees. Regarding *PhySIC_IST* 0, which mimics a voting approach, the number of nonidentical triplets between model tree and supertree, *d*, generally decreases with more input trees, whereas the number of star triplets in the supertree, *r*_1_, and the number of identical triplets, *s*, increase. In case of *PhySIC_IST*, *d* does not decrease as much as for *PhySIC_IST* 0, and can, as in case of 25% and 75%, also increase for more than ten input trees. Moreover, the number of star triplets increases and the number of identical triplets rather decreases in correspondence to the rather increasing value of *d* for the same number of input trees. For all deletion ratios, the voting-based BWD method produces less nonidentical triplets with a higher number of input trees. Star triplets are practically absent whereas the number of identical triplets clearly decreases for a higher number of input trees. The same behavior holds for the voting-based MRP method, although it produces a significantly higher number of star triplets, which decreases in case of 25% and 50%. To summarize, the voting based methods BWD SAC and MRP produce more identical triplets on the one hand (whereby the former produces practically no star triplets), but also high numbers of nonidentical triplets on the other hand when compared to *PhySIC_IST* 0. This behavior is generally counterbalanced by the veto-based methods by producing more star triplets.

Results for 48 Taxon Model Trees
See Figures 8 and 9.

Running Times for 525 Taxa Model Trees
See Table 6.
